# The Stress and Adversity Inventory for Adults (Adult STRAIN) in Korean: Initial Validation and Associations with Psychiatric Disorders

**DOI:** 10.3390/brainsci15010032

**Published:** 2024-12-30

**Authors:** Eun Soo Kim, Yun Tae Kim, Kang-Seob Oh, Young Chul Shin, Sang-Won Jeon, Dong-Won Shin, Sung Joon Cho, George M. Slavich, Junhyung Kim

**Affiliations:** 1 Department of Psychiatry, Kangbuk Samsung Hospital, Sungkyunkwan University School of Medicine, Seoul 03181, Republic of Korea; eunsoo421.kim@samsung.com (E.S.K.); kangseob.oh@samsung.com (K.-S.O.); yc523.shin@samsung.com (Y.C.S.); swj.jeon@samsung.com (S.-W.J.); down.shin@samsung.com (D.-W.S.); sungjoon.cho@samsung.com (S.J.C.); 2 Division of Biostatistics, Kangbuk Samsung Hospital, Seoul 03181, Republic of Korea; yt9771.kim@samsung.com; 3 Workplace Mental Health Institute, Kangbuk Samsung Hospital, Sungkyunkwan University School of Medicine, Seoul 03181, Republic of Korea; 4 Department of Psychiatry and Biobehavioral Sciences, University of California, Los Angeles, CA 90095, USA

**Keywords:** early adversity, childhood adversity, life stress, measurement, assessment, psychiatric disorders, Korean STRAIN

## Abstract

Background/Objectives: Stressors occurring across the life course are considered to have a cumulative impact on health, but there is no instrument for assessing lifetime stressor exposure in Korea. Therefore, we validated the Stress and Adversity Inventory (Adult STRAIN) in Korean. Methods: We translated the Adult STRAIN into Korean and examined its concurrent, predictive, and comparative predictive validity in 218 Korean adults (79 men, 139 women; *M*_age_ = 29.5; 19–50 years old) recruited from a psychiatric setting. We assessed concurrent validity using Pearson’s correlations, predictive validity using multiple regression models, and comparative predictive validity using multivariate logistic regression to identify participants with lifetime psychiatric diagnoses. Results: The Korean STRAIN exhibited sufficient usability and acceptability; good concurrent validity with other measures of early adversity, life events, and perceived stress (*r*s = 0.48–0.61); and strong predictive validity in relation to anxiety and depressive symptoms (β = 0.08–0.47; ΔR2 = 0.11–0.21). Each domain of Korean-style stress, based on the timing, type, life domain, and sociopsychological characteristics of stress exposure, showed a different distribution of lifetime psychiatric diagnosis probabilities (odds ratios = 1.20–4.85). Finally, the test–retest reliability for total lifetime stressor count and severity over four weeks was high. Conclusions: The Korean STRAIN is a practical, valid, and reliable instrument for researchers and clinicians to efficiently assess lifetime stressor exposure.

## 1. Introduction

The concept of “stress”, defined by Han Selye in 1936 as “the non-specific response of the body to any demand for change” [1], has evolved to encompass the body’s response to any mental, emotional, or physical disturbance. Stress has recently become a central concept that poses a significant threat to both mental and physical health [2,3,4]. Moreover, recent studies have suggested that humans are affected by stress throughout their entire lives, and the potential health risks associated with stress are vast [5,6,7].

A stressor is defined as any event, situation, or stimulus that triggers a stress response [8,9]. According to Lazarus and Folkman’s model, individuals appraise stressors and manage them through coping strategies, with failures in this process associated with adverse psychological and physiological outcomes [3,9,10,11]. Since Thomas Holmes developed life event lists linking stressors to illness [12], research has shown that different stressor domains, such as work-related or interpersonal stress, elicit unique responses [8,13,14,15]. For instance, interpersonal and work stress are often strongly associated with depression, whereas traumatic experiences such as combat and sexual violence greatly increase the risk for developing post-traumatic stress disorder (PTSD) [16,17,18,19]. Furthermore, stressor effects vary by the exposure timing and specific stressors experienced, with evidence showing that these dimensions influence many health outcomes, including telomere shortening, neurodevelopmental risks, long-term immune dysfunction, malignancy, and even early mortality [2,4,5,6,7,20]. Although a comprehensive understanding of these diverse stressor effects is thus crucial, significant gaps have existed with respect to our ability to assess these different features of stressors across the life course.

More specifically, few instruments that assess stressor exposure and its effects in high resolution have historically existed. The Perceived Stress Scale (PSS), probably the most widely used stress measure [21], is commonly used in large cohort studies examining how stress impacts mental and physical health [22,23]. Although the PSS provides a simple quantification of perceived stress levels over the past month, it does not assess stressor exposure and is highly sensitive to mood and personality effects. The Life Events Checklist for DSM-5 (LEC-5) is another widely used scale [23], particularly in PTSD-related research, as it assesses the experience of traumatic events [24]. However, the LEC-5 also does not provide information on stressor exposure timing or on the domains of stressors experienced. Childhood maltreatment and adversity are frequently measured using the Childhood Trauma Questionnaire-Short Form (CTQ-SF) [25] and the Adverse Childhood Experiences (ACE) questionnaire [26], which focus solely on specific early-life stressors, thus limiting their ability to evaluate adulthood stressors or the cumulative impact of stressors occurring across the life course. Indeed, a comprehensive and systemic tool for assessing multi-domain stressors across the lifespan has long been needed.

To address this critical measurement issue, in 2008, Slavich developed the Stress and Adversity Inventory for Adults (STRAIN) to assess lifetime stressor exposure [27]. The STRAIN is designed as a highly affordable, user-friendly, scalable, and reliable tool that can be self- or interviewer-administered in an online system. A particular strength of the STRAIN is that it has a multidimensional structure capable of evaluating lifetime stressor exposure. It assesses 55 different stressors through a total of 220 questions that evaluate stressor severity, frequency, timing, and duration. Using these raw data, it generates more than 115 summarized scores organized into multiple dimensions, including exposure indices (e.g., stressor count and severity), timing (e.g., early-life stress, adulthood life stress), types (e.g., acute life events and chronic difficulties), primary life domains (e.g., housing, education), and core social–psychological characteristics (e.g., interpersonal loss, physical danger). Therefore, the STRAIN can provide comprehensive information, including whether a specific event has been experienced, as measured by the LEC; details about childhood trauma experiences, assessed using the CTQ-SF; and insights into current stress levels, as measured by the PSS.

The STRAIN has now been translated into more than 30 languages, and validation studies of the German, English, and Brazilian versions of the STRAIN have demonstrated excellent usability and acceptability; test–retest reliability; concurrent, discriminant, and incremental validity; and predictive utility in relation to a wide variety of outcomes [27,28,29]. Moreover, STRAIN has been widely used to investigate the effect of cumulative exposure to specific lifetime stressors on psychological outcomes (e.g., anxiety, depression, burnout, well-being) and physiological health (e.g., cortisol reactivity, inflammation, biological aging), personality traits (e.g., ambiguity tolerance, reward sensitivity), and behaviors (e.g., alcohol use, risk behaviors) [30,31,32,33,34,35].

The development of psychiatric disorders is often associated with chronic exposure to various stressors, leading to increased allostatic load or cumulative “wear-and-tear” on the body [36]. Recurrent and excessive stress progressively weakens the body’s ability to maintain homeostasis and increases the risk of mental disorders, including depression, anxiety, neurodevelopmental disorders, and PTSD [4,37,38,39]. The STRAIN’s ability to evaluate a broad array of stressors that can exert these cumulative effects over the lifetime is expected to be crucial in helping to clarify associations between stressor exposure and psychiatric disorders. However, research on lifetime stressor exposure using the STRAIN and psychiatric disorders is limited, and despite its importance and multifaceted and cumulative design, no appropriate tool is available in Korea.

To address this issue, we first translated the STRAIN into Korean. Then, we examined the usability, acceptability, test–retest reliability, and concurrent, predictive, and incremental validity of the Adult STRAIN in Korean. While the current study has offered valuable preliminary data on the Korean STRAIN, our primary focus was on specific research questions rather than providing a comprehensive psychometric property. We hypothesized that the Adult STRAIN in Korean would demonstrate good usability/acceptability and test–retest reliability and be significantly correlated with other life stress measures. Furthermore, we hypothesized that cumulative lifetime stress exposure, as measured by the Korean STRAIN, would be associated with the lifetime psychiatric diagnosis but that these effects would vary by stressor type.

## 2. Materials and Methods

### 2.1. Participants and Procedure

Participants were 218 adults between 19 and 50 years old (79 men and 139 women) who were recruited through advertisements posted at the Korea University of Guro Hospital from June to November 2023. After providing informed consent, participants engaged in a survey of psychological factors (i.e., stress, anxiety, depression) and sociodemographic factors (e.g., demographics, medical history, and psychiatric history), including the Korean STRAIN.

Table 1 presents the participants’ characteristics. Participants in the initial survey had a mean age of 29.5 ± 6.02 years. A total of 171 participants (78.44%) in the sample were employed. Additionally, 66 participants reported a history of medical disorders, whereas 44 individuals reported a history of psychiatric disorders. Among these, depressive disorders were the most frequently reported, affecting 37 patients (16.97%). These were followed by anxiety disorder, reported by 31 patients (14.22%), and insomnia disorder, reported by 20 patients (9.17%). We re-administered the STRAIN to all participants approximately four weeks after enrollment (*M*_weeks_ = 5.33, *SD* = 3.20) to examine the test–retest reliability of the Korean STRAIN. In total, 177 participants completed the follow-up assessment. The study protocol was pre-approved by the Institutional Review Board of the Korea University Guro Hospital (IRB number: K2023-1381-001).

### 2.2. Measures

#### 2.2.1. Lifetime Stressor Exposure

To evaluate participants’ cumulative lifetime stressor exposure, we first translated the STRAIN into Korean using the gold-standard translation/back-translation method. The process involved three key steps: first, the original English STRAIN interview was translated into Korean; second, this Korean version of the STRAIN was translated back into English; finally, we carefully compared the original English STRAIN and the back-translated STRAIN to ensure accuracy and resolved any discrepancies via consensus discussion in order to confirm that the Korean STRAIN matched the English STRAIN. Critically, the first and second steps described above were carried out by two different, independent bilingual experts to ensure the accuracy and cultural appropriateness of the Korean STRAIN.

The resulting Korean STRAIN, similar to the original English version, encompasses an identical set of 55 core stressors [28], including acute life events (e.g., deaths of relatives) and chronic difficulties (e.g., ongoing health problems). The STRAIN provides participants with a set of “core” questions designed to determine whether they have encountered a certain stressor (e.g., “School and work occupy a significant part of many people’s lives. So, I’d like to ask you a few questions about your school and work history. Have you ever dropped out or failed out of school?”). If the participant responds with “Yes” to signify that they have experienced certain stressors, they will next answer questions on the frequency of such occurrences in their life, the subjective severity of the experience, and the timing of these events. If the event has occurred several times in their life, they will receive additional follow-up questions about the multiple occurrences. If a participant has not encountered a stressor, the STRAIN’s branching logic will bypass the follow-up questions and go to the subsequent core question. This comprehensive approach enabled us to create highly nuanced and individualized lifetime stressor exposure profiles for each participant while also synthesizing the data into various aggregate measures of lifetime stressor exposure. In this study, we focused on the STRAIN’s two primary outcomes: the (a) total lifetime stressor count, i.e., the aggregate number of stressors experienced, and (b) total lifetime stressor severity, i.e., their cumulative severity over each participant’s entire lifetime. This approach replicated the procedure followed in the original, German, and Brazilian Portuguese Adult STRAIN validation studies [27,28,29].

#### 2.2.2. Early Adversity

Early-life adversity was evaluated using the Korean version of the CTQ-SF [26]. This 28-item measure assesses five dimensions of childhood maltreatment, including physical and emotional neglect and sexual, physical, and emotional abuse (e.g., “I had to wear dirty clothes”). Responses range from 1 (never true) to 5 (very often true), with higher average scores indicating greater early adversity. The Korean CTQ-SF has demonstrated high internal consistency in prior research (Cronbach’s α = 0.88) and excellent reliability in the present study (Cronbach’s α = 0.93, McDonald’s ω = 0.95) [26].

#### 2.2.3. Life Events

Exposure to potentially traumatic life events was measured using the Korean version of the LEC-5 [24]. This self-report instrument screens for 16 events known to potentially result in PTSD or significant distress. Respondents indicate whether each event “happened to me”, was “witnessed”, or “does not apply”. The total number of directly experienced events provides an index of life event exposure. The Korean LEC-5’s reliability improved over a prior study (current Cronbach’s α = 0.85, McDonald’s ω = 0.88, compared to 0.67 of Cronbach’s α in prior research) [24].

#### 2.2.4. Perceived Stress

Recent perceived stress levels were assessed using the Korean PSS [21]. Participants reported ten different stress experiences over the past month using a five-point Likert scale, with higher scores indicating greater perceived stress (e.g., “How often have you felt startled or upset because of something unexpected?”). The Korean PSS maintained good reliability (Cronbach’s α = 0.85, McDonald’s ω = 0.91), consistent with prior findings (Cronbach’s α = 0.82) [21].

#### 2.2.5. Anxiety and Depressive Symptoms

The Korean State–Trait Anxiety Inventory (STAI) [40,41] assessed both state (current) and trait (general) anxiety using 40 items rated on a four-point scale (e.g., Trait: “I make decisions easily.”, State: “I am tense.”). Both subscales demonstrated excellent reliability (Cronbach’s α = 0.95 and 0.94 for state and trait, respectively).

The Korean 7-item Generalized Anxiety Disorder scale (GAD-7) [42] evaluated symptoms of generalized anxiety over the past two weeks (e.g., “I feel nervous, anxious, or on edge.”). It showed high internal consistency (Cronbach’s α = 0.90, McDonald’s ω = 0.89), similar to prior findings (Cronbach’s α = 0.93) [42].

Depressive symptoms were assessed using the Korean Patient Health Questionnaire-9 (PHQ-9) [43]. This nine-item measure assesses depressive symptoms over the past two weeks using a four-point Likert scale (e.g., “I felt down, depressed, or hopeless.”). The Korean PHQ-9 maintained excellent reliability (Cronbach’s α = 0.91, McDonald’s ω = 0.91) compared to prior research (Cronbach’s α = 0.88) [43].

### 2.3. Data Analyses

To analyze concurrent validity, we examined Pearson’s correlations between the Korean STRAIN and psychological factors, including CTQ-SF, LEC-5, and PSS scores. Predictive validity was assessed using multiple regression models by evaluating how well the Korean STRAIN predicts participants’ anxiety and depressive symptoms, measured by the STAI trait and state, GAD-7, and PHQ-9 scores. Additionally, we conducted multivariate logistic regression analyses to evaluate the comparative predictive validity of the Korean STRAIN to distinguish between participants with and without psychiatric disorders, examining the likelihood of diagnosis based on timing, type, domain, and core sociopsychological characteristics. All multiple regression models included the following covariates: age, sex, education level, and occupation. The test–retest reliability of the Korean STRAIN was evaluated using Pearson’s correlation coefficient. We applied Student’s *t*-tests to examine sex differences in specific primary life domains and core sociopsychological characteristics of lifetime stressor count. All analyses were conducted using R software (version 4.4.1; R Foundation for Statistical Computing, Vienna, Austria).

## 3. Results

### 3.1. Usability and Acceptability

The Korean STRAIN assessment demonstrated efficient completion times across both administrations. During the initial assessment, participants completed the Korean STRAIN in a median time of 16 min (interquartile range [IQR] = 10 min–22 min). The retest showed improved efficiency, with a median completion time of 12 min (IQR = 8 min–17 min). The instrument exhibited remarkable acceptability among the study cohort. Indeed, all participants successfully completed the entire interview with no instances of premature termination. Furthermore, no participants reported any complaints or psychosocial distress stemming from engagement with the questions.

### 3.2. Descriptive Statistics for Lifetime Stressor Exposure for Men and Women

On average, participants reported 8.53 stressors over their life course (SD = 9.90; range 0–55). Table 2 presents our main variable of interest distribution, means, standard deviations, skewness, and kurtosis data. The skewness values were within the acceptable range for normality. Although the kurtosis values encompassed the acceptable range for normality, our sample size was large enough to consider the Central Limit Theorem (>200), so we were able to assume a normal distribution and perform the analysis.

Table 3 provides an analysis of lifetime stressor counts by sex. Women experienced more stressors (M = 11.46, SD = 11.63) than men (M = 8.68, SD = 6.61). This disparity was especially pronounced for stressors involving the primary life domains of housing, reproduction, and life-threatening situations (all *p* < 0.05). Furthermore, examining the STRAIN’s core sociopsychological characteristics revealed sex-based differences that were particularly pronounced for stressors involving physical danger and role change/disruption (all *p* < 0.05, Table 3).

### 3.3. Validity

#### 3.3.1. Concurrent Validity

To evaluate the validity of the Korean STRAIN, we first investigated its concurrent validity by comparing it with other commonly used stress assessment tools [44]. Our analysis focused on associations between the Korean STRAIN and three well-established stress measures: CTQ-SF for assessing early-life adversity, LEC-5 for assessing significant life events, and PSS for assessing recent perceived stress levels.

The results revealed significant correlations between the Korean STRAIN’s two main outcomes—lifetime stressor count and lifetime stressor severity—and these commonly used, well-validated instruments. Indeed, both STRAIN indices demonstrated very strong, positive associations with childhood trauma as measured by the CTQ-SF (count: *r* = 0.614, *p* < 0.05; severity: *r* = 0.604, *p* < 0.05). Comparable patterns were observed with life events assessed using the LEC-5 (count: *r* = 0.487, *p* < 0.05; severity: *r* = 0.473, *p* < 0.01). Moreover, STRAIN metrics showed robust correlations with perceived stress levels quantified using the PSS (count: *r* = 0.477, *p* < 0.05; severity: *r* = 0.509, *p* < 0.05). Table 4 presents these correlation coefficients in detail.

#### 3.3.2. Predictive Validity

Subsequently, we evaluated the predictive validity of the STRAIN by investigating its ability to predict participants’ levels of anxiety and depressive symptoms experienced within the previous two weeks [45]. Multiple separate regression models were used to examine the extent to which the Korean STRAIN predicted participants’ anxiety and depressive symptoms, as assessed using the STAI trait and state, GAD-7, and PHQ-9.

As shown in Table 5, the total lifetime stressor count was significantly associated with the STAI state scale (β = 0.44; ΔR2 = 0.120, *p* < 0.001), STAI trait scale (β = 0.47; ΔR2 = 0.117, *p* < 0.001), PHQ-9 scores (β = 0.27; ΔR2 = 0.182, *p* < 0.001), and GAD-7 scores (β = 0.17; ΔR2 = 0.109, *p* < 0.001). Similar results were found for models assessing lifetime stressor severity, wherein the total lifetime stressor severity was significantly associated with the STAI state scale (β = 0.20; ΔR2 = 0.136, *p* < 0.001), STAI trait scale (β = 0.23; ΔR2 = 0.150, *p* < 0.001), PHQ-9 scores (β = 0.12; ΔR2 = 0.206, *p* < 0.001), and GAD-7 scores (β = 0.08; ΔR2 = 0.121, *p* < 0.001).

#### 3.3.3. Comparative Predictive Validity of Each Variable for Lifetime Psychiatric Diagnosis

**Figure 1:**
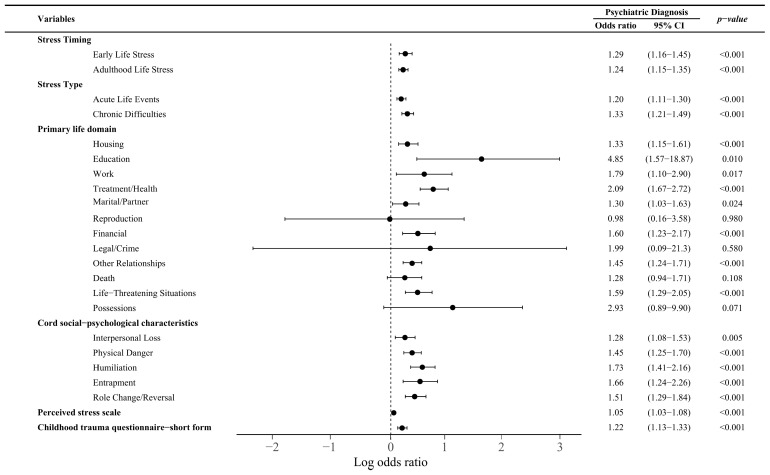
Likelihood of lifetime psychiatric diagnosis by stressor timing, type, primary domain, and core sociopsychological characteristic of the STRAIN, PSS, and CTQ-SF. CTQ-SF, Childhood Trauma Questionnaire–Short Form; PSS, Perceived Stress Scale; STRAIN, Stress and Adversity Inventory for Adults.

To compare predictive validity of each domain of the STRAIN and other commonly used stress assessment tools for lifetime psychiatric diagnosis [45], we analyzed the likelihood of diagnosis based on the stressor timing, type, domains, and sociopsychological characteristics of the STRAIN, PSS, and CTQ-SF (Figure 1) [46].

For stressor timing and stressor type, the odds ratios were high for early-life stressors (odds ratio [OR] = 1.29, 95% confidence interval [CI] = 1.16–1.45, *p* < 0.001) and chronic difficulties (OR = 1.33, 95% CI = 1.21–1.49, *p* < 0.001) vs. adulthood stressors (OR = 1.24, 95% CI = 1.15–1.35, *p* < 0.001) and acute life events (OR = 1.20, 95% CI = 1.11–1.30, *p* < 0.001), respectively. In terms of the primary life domains, education (OR = 4.85, 95% CI = 1.57–18.87, *p* = 0.010), treatment/health (OR = 2.09, 95% CI = 1.67–2.72, *p* < 0.001), and work (OR = 1.79, 95% CI = 1.10–2.90, *p* < 0.001) presented relatively high ORs. Regarding core sociopsychological characteristics, humiliation (OR = 1.73, 95% CI = 1.41–2.16, *p* < 0.001) and entrapment (OR = 1.66, 95% CI = 1.24–2.26, *p* < 0.001) were significantly associated with increased odds of being diagnosed with a lifetime psychiatric disorder. PSS (OR = 1.05, 95% CI = 1.03–1.08, *p* < 0.001) and CTQ-SF (OR = 1.22, 95% CI = 1.13–1.33, *p* < 0.001) scores were also significant.

### 3.4. Test–Retest Reliability

Finally, we assessed the test–retest reliability of all six of the Korean STRAIN’s main outcomes (i.e., total lifetime stressor count, total lifetime stressor severity, acute life event count, chronic difficulty count, acute life event severity, chronic difficulty severity) over a one-month period. All six of these outcomes exhibited very high test–retest reliability (*r* ≥ 0.878, *p* < 0.05) over one month. Among them, total lifetime stressor count and total lifetime stressor severity achieved outstanding test–retest reliability over time (*r* = 0.910, *p* < 0.05, and *r* = 0.909, *p* < 0.05, respectively) (Table S1).

## 4. Discussion

This study is the first to investigate the usability and acceptability, reliability, and validity of the Korean STRAIN, as well as how the various stressor dimensions assessed by the STRAIN relate to participants’ lifetime psychiatric diagnosis. Participants completed the Korean STRAIN in approximately 16 min in the first session and 12 min in the second session, with excellent overall usability and acceptability. The Korean STRAIN demonstrated strong concurrent validity with the CTQ-SF, LEC-5, and PSS, as well as excellent predictive validity with the STAI-Trait, STAI-State, GAD-7, and PHQ.

An analysis of the concurrent and predictive validity of the Korean STRAIN revealed strong correlations between the STRAIN scores and other scales assessing early adversity, adulthood life events, and recent perceived stress levels. Consistent with prior research examining the original Adult STRAIN (in English) and German Adult STRAIN, the CTQ-SF showed the strongest correlation with lifetime stressor count and severity [29], underscoring the critical role of early developmental stages in processing stressor exposure [47,48,49]. By capturing participants’ cumulative stressors from an early age, the Korean STRAIN provides a highly nuanced, comprehensive view of individuals’ lifelong stressor burden. Moreover, the STRAIN scores significantly predicted trait and state anxiety, as well as self-reported anxiety and depressive symptoms, supporting its strong predictive validity in relation to several key clinical outcomes. These findings are consistent with numerous prior studies reporting the onset of psychopathologies such as depression and anxiety following recent major life events and trauma [4,9,50,51,52], and the robust correlations between the Korean STRAIN and mental health highlight its potential utility for use assessing stress in clinical settings.

The findings of this study show that the STRAIN in Korean is consistent with prior studies of other stress assessments in relation to lifetime psychiatric diagnosis. Specifically, the data showed that chronic childhood stressors were associated with a significantly elevated risk of lifetime psychiatric diagnosis, consistent with prior studies demonstrating that early-life stress, measured using the ACE questionnaire, CTQ-SF, and clinical interviews, increases the risk of developing conditions such as PTSD, depression, and conduct disorders [6,33,53,54]. The primary life domain results reflect Korean society’s characteristics, where academic stress leads to life dissatisfaction, highlighting prominent features in the educational domain [55,56].

Additionally, this study identified humiliation and entrapment as dimensions of stress that are highly relevant for the development of psychiatric disorders [57,58,59], entrapment and defeat theory [60], social rank theory [61], and the interpersonal theory of suicide [59]. Although many models have hypothesized an association between specific categories of life stressors and psychopathology, few studies have comprehensively assessed the social characteristics of stressors, especially across the life course. By capturing the multidimensional characteristics and cumulative effects of psychological stressors, the Korean STRAIN could be highly beneficial to those looking to develop or refine theories of psychopathology and other health outcomes.

Finally, we found that the Korean STRAIN has excellent test–retest reliability over one month, even though achieving high test–retest reliability of a stressor scale requires participants to accurately (re-)remember not just which stressors they have experienced but also the specific severity, frequency, exposure timing, duration, and type (acute vs. chronic) of those stressors. Regardless, the test–retest reliability for the Korean STRAIN’s six main outcomes was very high, with total lifetime stressor count and total lifetime stressor severity exhibiting adequate test–retest reliability at *r* = 0.910 and *r* = 0.909, respectively. These values are remarkably comparable to those observed for the original (English) Adult STRAIN (*r* = 0.904 and 0.919, respectively) [26] and the Brazilian Adult STRAIN (*r* = 0.936 and 0.953, respectively) [25], demonstrating the ability of the STRAIN to obtain consistent assessments of individuals’ lifetime stressor exposure over time.

### Limitations

This study had several limitations. First, the primarily cross-sectional study design (except for the test–retest analyses) means that all findings are correlational, and causality cannot be inferred. Second, although it has been shown that the score is relatively robust in the face of mood and self-report biases [29], the STRAIN is based on participants’ self-reports, and these biases cannot be fully ruled out. Third, no biological samples were collected in this study; therefore, further research is needed to validate the STRAIN against health-related biomarkers. Fourth, the sample was drawn from a specific region in Korea and consisted of individuals with access to psychiatric care in the country, which may limit the generalizability of the findings. Finally, due to the limited sample size, limited information was available regarding the onset of specific clinical conditions. Future studies should evaluate the generalizability of the associations described here in more representative populations and specific clinical samples (e.g., anxiety, depression, cognitive decline, substance use).

## 5. Conclusions

In conclusion, the Korean STRAIN is a user-friendly and highly acceptable tool for assessing lifetime stressor exposure that demonstrates excellent concurrent, predictive, and comparative predictive validity and outstanding test–retest reliability over one month. The Korean STRAIN’s multidimensional and cumulative nature could make it particularly useful for comprehensively assessing lifetime stressors to aid in case conceptualization and treatment planning in clinical settings. The Korean STRAIN may also help to identify individuals at high risk for psychiatric disorders, supporting the delivery of prevention programs for promoting resilience in high-risk individuals.

## Figures and Tables

**Table 1:** Participant characteristics.

Characteristic		Total (*N* = 218)
Age (years)		29.5 ± 6.02
Sex		
	Male	79 (36.24)
	Female	139 (63.76)
Level of education (years)		14.90 ± 1.73
Occupational status		
	Employed	171 (78.44)
	Unemployed	47 (21.56)
Medical history		
	Any	66 (30.28)
	Endocrine disorder	24 (11.01)
	Gastrointestinal disorder	23 (10.55)
	Nervous disorder	6 (2.75)
	Musculoskeletal disorder	6 (2.75)
	Respiratory disorder	5 (2.29)
	Genitourinary disorder	4 (1.83)
	Cardiovascular disorder	3 (1.38)
	Others	7 (3.21)
	None	152 (69.72)
Psychiatric history		
	Any	44 (20.33)
	Depressive disorder	37 (16.97)
	Anxiety disorder	31 (14.22)
	Insomnia disorder	20 (9.17)
	Panic disorder	7 (3.21)
	Obsessive compulsive disorder	2 (0.92)
	Bipolar disorder	1 (0.46)
	Adjustment disorder	1 (0.46)
	Post-traumatic stress disorder	0 (0.00)
	Schizophrenia	0 (0.00)
	Others	1 (0.46)
	None	174 (79.66)

Data are presented as mean ± standard deviation or proportion (%).

**Table 2:** Distribution and descriptive statistics of the STRAIN.

	Mean	SD	*sk*	*ku*
Total Lifetime Stressor Count	10.45	10.17	1.86	4.28
Total Lifetime Stressor Severity	25.17	25.07	1.79	3.94
Acute Life Event Count	5.6	6.1	2.29	6.35
Chronic Difficulty Count	4.86	5.1	1.25	1.07
Acute Life Event Severity	10.31	10.53	1.88	4.54
Chronic Difficulty Severity	14.86	16.34	1.56	2.70

SD, standard deviation; sk, skewness; ku, kurtosis.

**Table 3:** Lifetime stressor count by stressor category for men and women.

Variables		Sex		*Cohen’s D* (*p*-Value)
		Men (*n* = 79)	Women (*n* = 139)	
Total Lifetime Stressor Count		8.68 ± 6.61	11.46 ± 11.63	−0.27 (0.052)
Primary Life Domain				
	Housing	0.65 ± 1.34	1.40 ± 2.77	−0.32 (0.024 *)
	Education	0.06 ± 0.29	0.04 ± 0.19	0.12 (0.402)
	Work	0.43 ± 0.63	0.60 ± 0.83	−0.23 (0.108)
	Treatment/Health	1.49 ± 2.19	1.55 ± 2.01	−0.03 (0.856)
	Marital/Partner	1.43 ± 1.53	1.42 ± 2.00	0.01 (0.960)
	Reproduction	0.00 ± 0.00	0.12 ± 0.42	−0.34 (0.015 *)
	Financial	0.38 ± 0.74	0.68 ± 1.25	−0.27 (0.056)
	Legal/Crime	0.06 ± 0.29	0.01 ± 0.12	0.24 (0.084)
	Other Relationships	1.70 ± 2.13	2.09 ± 2.54	−0.17 (0.241)
	Death	0.96 ± 1.32	0.76 ± 1.16	0.16 (0.246)
	Life-Threatening Situations	0.65 ± 1.39	1.32 ± 2.53	−0.31 (0.030 *)
	Possessions	0.03 ± 0.16	0.08 ± 0.30	−0.21 (0.136)
Core Social–Psychological Characteristic				
	Interpersonal Loss	2.84 ± 2.18	2.61 ± 2.21	0.10 (0.471)
	Physical Danger	1.51 ± 2.02	2.38 ± 3.26	−0.30 (0.032 *)
	Humiliation	1.25 ± 1.83	1.47 ± 2.00	−0.11 (0.433)
	Entrapment	0.97 ± 1.07	1.22 ± 1.27	−0.21 (0.144)
	Role Change/Disruption	1.65 ± 2.05	2.81 ± 4.32	−0.32 (0.025 *)

Data are presented as mean ± standard deviation. * *p* < 0.05.

**Table 4:** Zero-order correlation between STRAIN indices and scales assessing early adversity, life events, and recent perceived stress.

		*M* ± *SD*	1	2	3	4	5
1	STRAIN Lifetime Stressor Count	10.45 ± 10.17		0.95 *	0.61 *	0.49 *	0.48 *
2	STRAIN Lifetime Stressor Severity	25.17 ± 25.07			0.60 *	0.47 *	0.51 *
3	CTQ-SF	39.42 ± 14.33				0.33 *	0.47 *
4	LEC	1.66 ± 2.32					0.22 *
5	PSS	17.16 ± 5.91					

*M*, mean; *SD*, standard deviation; CTQ-SF, Childhood Trauma Questionnaire–Short Form; LEC-5, Life Events Checklist for DSM-5; PSS, Perceived Stress Scale; STRAIN, Stress and Adversity Inventory for Adults. Total *N* = 218. * *p* < 0.05.

**Table 5:** Multiple regression models parameters for the predictive validity for the STRAIN with the subscales of the STAI and depressive and anxiety symptom levels.

	**STAI State**					
**Model**	***β***	***SE***	**Adj.R2**	**ΔR2**	***F***	***p***
Covariates	-	9.64	0.23		13.97	<0.001
Covariates + STRAIN Total Stressor Count	0.44 ***	8.86	0.35	0.12	20.47	<0.001
Covariates + STRAIN Total Stressor Severity	0.20 ***	8.75	0.37	0.136	21.87	<0.001
	**STAI Trait**					
	***β***	***SE***	**Adj.R2**	**ΔR2**	***F***	***p***
Covariates	-	10.39	0.24		14.84	<0.001
Covariates + STRAIN Total Stressor Count	0.47 ***	9.56	0.36	0.117	21.25	<0.001
Covariates + STRAIN Total Stressor Severity	0.23 ***	9.31	0.39	0.15	24.28	<0.001
	**PHQ-9**					
	***β***	***SE***	**Adj.R2**	**ΔR2**	***F***	***p***
Covariates	-	4.85	0.22		12.92	<0.001
Covariates + STRAIN Total Stressor Count	0.27 ***	4.25	0.4	0.182	24.89	<0.001
Covariates + STRAIN Total Stressor Severity	0.12 ***	4.17	0.42	0.206	27.35	<0.001
	**GAD-7**					
	***β***	***SE***	**Adj.R2**	**ΔR2**	***F***	***p***
Covariates	-	4.25	0.12		6.84	<0.001
Covariates + STRAIN Total Stressor Count	0.17 ***	3.98	0.23	0.109	11.64	<0.001
Covariates + STRAIN Total Stressor Severity	0.08 ***	3.94	0.24	0.121	12.39	<0.001

Covariates: age, sex, level of education, occupation. STAI, State and Trait Anxiety Inventory; PHQ-9, Patient Health Questionaire-9; GAD-7, 7-item Generalized Anxiety Disorder; STRAIN, Stress and Adversity Inventory for Adults. *** *p* < 0.001.

## Data Availability

The data presented in this study are available on request from the corresponding author. The data are not publicly available due to ethical restrictions.

## Supplementary Materials

The following supporting information can be downloaded at https://www.mdpi.com/article/10.3390/brainsci15010032/s1. Table S1. Test–retest reliability of the core 6 items of the STRAIN.
