# Supplementary material for: The Stress and Adversity Inventory for Adults (Adult STRAIN) in Korean: Initial Validation and Associations with Psychiatric Disorders
Source: Brain Sci. 2024 Dec 30;15(1):32. doi: 10.3390/brainsci15010032 (PMC11763812; doi:10.3390/brainsci15010032)
Supplement: Supplementary file 1 [file brainsci-15-00032-s001.zip › Supplementary Table S1.pdf]

brain sciences

MDPI

Supplementary Material

Table S1. Test-retest reliability of the primary six outcomes of the Korean STRAIN

	**Test-retest reliability (Pearson's *r*)**
Total Lifetime Stressor Count	0.91*
Total Lifetime Stressor Severity	0.91*
Acute Life Event Count	0.88*
Chronic Difficulty Count	0.88*
Acute Life Event Severity	0.89*
Chronic Difficulty Severity	0.88*

Total $N = 177$

* $p < 0.05$
